# The Intergroup Rhabdomyosarcoma Study Group (IRSG): Major Lessons From the IRS-I Through IRS-IV Studies as Background for the Current IRS-V Treatment Protocols

**DOI:** 10.1080/13577140120048890

**Published:** 2001-03

**Authors:** R. Beverly Raney, Harold M. Maurer, James R. Anderson, Richard J. Andrassy, Sarah S. Donaldson, Stephen J. Qualman, Moody D. Wharam, Eugene S. Wiener, William M. Crist

**Affiliations:** 1 Department of Clinical Pediatrics UT MD Anderson Cancer Center Houston Texas USA; 2 The Office of the Chancellor University of Nebraska Medical Center Omaha Nebraska USA; 3 The Department of Preventive and Societal Medicine University of Nebraska Medical Center Omaha Nebraska USA; 4 Department of Surgery UT MD Anderson Cancer Center Houston Texas USA; 5 The Department of Radiation Oncology Stanford University Medical Center Stanford California USA; 6 Department of Laboratory Medicine Columbus Children's Hospital Columbus Ohio USA; 7 Johns Hopkins Oncology Center Baltimore Maryland USA; 8 The Department of Pediatric Surgery Pittsburgh Children's Hospital Pittsburgh Pennsylvania USA; 9 Office of the Dean University of Missouri School of Medicine Columbia Missouri USA; 10 Department of Pediatrics UT MD Anderson Cancer Center Box 87 1515 Holcombe Boulevard Houston TX 77030 USA

## Abstract

*Purpose.* To enumerate lessons from studying 4292 patients with rhabdomyosarcoma (RMS) in the Intergroup Rhabdomyosarcoma Study Group (IRSG, 1972–1997).

*Patients.* Untreated patients < 21 years of age at diagnosis received systemic chemotherapy, with or without irradiation (XRT) and/or surgical removal of the tumor.

*Methods.* Pathologic materials and treatment were reviewed to ascertain compliance and to confirm response and relapse status.

*Results.* Survival at 5 years increased from 55 to 71% over the period. Important lessons include the fact that extent of disease at diagnosis affects prognosis. Re-excising an incompletely removed tumor is worthwhile if acceptable form and function can be preserved. The eye, vagina, and bladder can usually be saved. XRT is not necessary for children with localized, completely excised embryonal RMS. Hyperfractionated XRT has thus far not produced superior local control rates compared with conventional, once-daily XRT. Patients with non-metastatic cranial parameningeal sarcoma can usually be cured with localized XRT and systemic chemotherapy, without whole-brain XRT and intrathecal drugs. Adding doxorubicin, cisplatin, etoposide, and ifosfamide has not significantly improved survival of patients with gross residual or metastatic disease beyond that achieved with VAC (vincristine, actinomycin D, cyclophosphamide) and XRT. Most patients with alveolar RMS have a tumor-specific translocation. Mature rhabdomyoblasts after treatment of patients with bladder rhabdomyosarcoma are not necessarily malignant, provided that the tumor has shrunk and malignant cells have disappeared.

*Discussion.* Current IRSG-V protocols, summarized herein, incorporate recommendations for risk-based management. Two new agents, topotecan and irinotecan, are under investigation for patients who have an intermediate or high risk of recurrence.


*Sarcoma* (2001) **5**, 9–15

ORIGINAL ARTICLE

### The Intergroup Rhabdomyosarcoma Study Group (IRSG): major lessons from the IRS-I through IRS-IV studies as background for the current IRS-V treatment protocols

R. BEVERLY RANEY,[1] HAROLD M. MAURER,[2] JAMES R. ANDERSON,[3] RICHARD J. ANDRASSY,[4] SARAH S. DONALDSON,[5] STEPHEN J. QUALMAN,[6] MOODY D. WHARAM,[7] EUGENE S. WIENER[8] & WILLIAM M. CRIST,[9] FOR THE IRSG REPRESENTING THE CHILDREN'S CANCER GROUP, THE PEDIATRIC ONCOLOGY GROUP, AND THE INTERGROUP RHABDOMYOSARCOMA STATISTICAL OFFICE*

*[1]Department of Clinical Pediatrics, and [4]Department of Surgery, UT MD Anderson Cancer Center, Houston, Texas, USA, [2]The Office of the Chancellor, and [3]The Department of Preventive and Societal Medicine, University of Nebraska Medical Center, Omaha, Nebraska, USA, [5]The Department of Radiation Oncology, Stanford University Medical Center, Stanford, California, USA, [6]Department of Laboratory Medicine, Columbus Children's Hospital, Columbus, Ohio, USA, [7]Johns Hopkins Oncology Center, Baltimore, Maryland, USA, [8]The Department of Pediatric Surgery, Pittsburgh Children's Hospital, Pittsburgh, Pennsylvania, USA, and the [9]Office of the Dean, University of Missouri School of Medicine, Columbia, Missouri, USA*

**Abstract**

*Purpose*. To enumerate lessons from studying 4292 patients with rhabdomyosarcoma (RMS) in the Intergroup Rhabdomyosarcoma Study Group (IRSG, 1972–1997).

*Patients*. Untreated patients < 21 years of age at diagnosis received systemic chemotherapy, with or without irradiation (XRT) and/or surgical removal of the tumor.

*Methods*. Pathologic materials and treatment were reviewed to ascertain compliance and to confirm response and relapse status.

*Results*. Survival at 5 years increased from 55 to 71% over the period. Important lessons include the fact that extent of disease at diagnosis affects prognosis. Re-excising an incompletely removed tumor is worthwhile if acceptable form and function can be preserved. The eye, vagina, and bladder can usually be saved. XRT is not necessary for children with localized, completely excised embryonal RMS. Hyperfractionated XRT has thus far not produced superior local control rates compared with conventional, once-daily XRT. Patients with non-metastatic cranial parameningeal sarcoma can usually be cured with localized XRT and systemic chemotherapy, without whole-brain XRT and intrathecal drugs. Adding doxorubicin, cisplatin, etoposide, and ifosfamide has not significantly improved survival of patients with gross residual or metastatic disease beyond that achieved with VAC (vincristine, actinomycin D, cyclophosphamide) and XRT. Most patients with alveolar RMS have a tumor-specific translocation. Mature rhabdomyoblasts after treatment of patients with bladder rhabdomyosarcoma are not necessarily malignant, provided that the tumor has shrunk and malignant cells have disappeared.

*Discussion*. Current IRSG-V protocols, summarized herein, incorporate recommendations for risk-based management. Two new agents, topotecan and irinotecan, are under investigation for patients who have an intermediate or high risk of recurrence.

**Key words**: *rhabdomyosarcoma, undifferentiated sarcoma, childhood/adolescence, IRS-V Protocol*

### Introduction

Soft-tissue sarcomas comprise the fifth most common type of childhood solid tumor, and rhabdomyosarcoma (RMS) is the most common form encountered in the first two decades of life. The disease can arise at any site and in any tissue in the body except bone. There are several histologic

Correspondence to: R. Beverly Raney, MD, Department of Pediatrics, UT MD Anderson Cancer Center, Box 87, 1515 Holcombe Boulevard, Houston, TX 77030, USA. Tel: (+1) 713-792-6624; E-mail: Braney@mdanderson.org

*Including James R. Anderson, PhD, Richard J. Andrassy, MD, Carola A.S. Arndt, MD, K. Scott Baker, MD, Frederic G. Barr, MD, PhD, W. Archie Bleyer, MD, Philip Breitfeld, MD, John C. Breneman, MD, Julia Bridge, MD, Kenneth Brown, MD, William M. Crist, MD, Sarah S. Donaldson, MD, Holcombe E. Grier, MD, Douglas Hawkins, MD, Peter J. Houghton, PhD, Michael Link, MD, Thom L. Lobe, MD, Harold M. Maurer, MD, William H. Meyer, MD, Jeff Michalski, MD, Sharon Murphy, MD, Charles N. Paidas, MD, Alberto S. Pappo, MD, David M. Parham, MD, Stephen J. Qualman, MD, R. Beverly Raney, MD, Leslie Robison, PhD, Eric Sandler, MD, Lynn Smith, MD, Poul H.B. Sorensen, MD, PhD, Sheri L. Spunt, MD, Lisa Teot, MD, Timothy Triche, MD, Teresa J. Vietti, MD, David Walterhouse, MD, Moody Wharam, MD, Eugene S. Wiener, MD, Suzanne Wolden, MD & Richard Womer, MD.

1357–714X print/1369–1643 online/01/010009-07 © 2001 Taylor & Francis Ltd
DOI: 10.1080/13577140120048890

subtypes: embryonal RMS (ERMS), the botryoid and spindle-cell variants of ERMS, and alveolar RMS (ARMS). ERMS is approximately three times more frequent than ARMS. In addition, RMS can metastasize to any tissue or organ in the body. All of these features lead to a myriad of forms of the disease, rendering it difficult to classify patients into homogeneous groups.

The Intergroup Rhabdomyosarcoma Study Group (IRSG) was formed under the auspices of the National Cancer Institute in 1972 to investigate the therapy and biology of RMS and undifferentiated sarcoma (UDS) in previously untreated patients less than 21 years of age. The patients were recruited from member institutions of the three cooperative pediatric cancer treatment groups existing at the time. Since then, five successive clinical protocols involving 4292 eligible patients have been completed: IRS-I, 1972–1978; IRS-II, 1978–1984; IRS-III, 1984–1991, IRS-IV Pilot (for patients with advanced disease only), 1987–1991; and IRS-IV, 1991–1997.[1–7] Many of the several trials conducted within each protocol were randomized. In addition, the accumulation of a large number of cases of relatively uncommon tumors has led to acquisition of important new information about the evolution of the disease and its biology.

The purposes of this article are: (1) to summarize the important lessons learned from the IRSG protocols over the past 25 years; and (2) to outline the current therapeutic approaches for newly diagnosed patients who may be eligible for treatment on the IRS-V study. IRS-V was opened in 1997 for patients with low-risk disease (i.e. with a good prognosis for survival), and subsequently in 1999 for the other patients.

### Patients and methods

### *Grouping and staging*

Patients are separated into four groups based on the extent of the disease as determined by clinical and radiographic imaging studies, along with a sample of bone marrow and tissue for pathologic examination taken from the primary tumor site. A cerebrospinal fluid sample is required for patients with cranial parameningeal tumors. Table 1 presents the surgical–pathologic grouping system, which categorizes patients according to the extent of disease remaining after the initial surgical procedure(s) but before beginning chemotherapy and radiation therapy (XRT). During the evolution of the IRSG protocols, it became apparent that there was a need to adopt a pre-clinical staging system that did not depend on the surgeon's decision of how much tissue to remove or on pathologic assessment of the specimen. The staging system was developed as a modified tumor–node–metastasis system, similar to classifications used by the International Union Against Cancer (UICC) and by our European colleagues.[8]

Table 2 displays this staging system, which separates patients by site of the primary tumor, tumor size, and the presence or absence of tumor-involved regional lymph nodes and of distant metastases. Currently, the staging and grouping systems and the tumor histologic subtype are all used to make decisions about treatment. Patients are placed into categories according to the prediction of survival, using the staging system and histologic subtype; various combinations of chemotherapeutic agents are administered accordingly. The grouping system categorizes patients by the amount of residual disease after initial surgery; XRT is administered according to each patient's group and histologic subtype.

**Table 1**. *IRSG surgical–pathologic grouping system*

| Group | Definition |
|---|---|
| I | Localized tumor, completely removed with pathologically clear margins and no regional lymph node involvement |
| II | Localized tumor, grossly removed with (a) microscopically involved margins, (b) involved, grossly resected regional lymph nodes, or (c) both |
| III | Localized tumor, with gross residual disease after grossly incomplete removal, or biopsy only |
| IV | Distant metastases present at diagnosis |

**Table 2**. *IRSG staging system*

| Stage | Sites of primary tumor | Tumor size (cm) | Regional lymph nodes | Distant metastases |
|---|---|---|---|---|
| 1 | Orbit, non-PM head/neck; GU non-bladder/prostate; biliary tract | Any size | N0, N1 | M0 |
| 2 | All other sites | $\leq 5$ | N0 | M0 |
| 3 | All other sites | $\leq 5$ | N1 | M0 |
| | | $> 5$ | N0 or N1 | |
| 4 | Any site | Any size | N0 or N1 | M1 |

PM, Parameningeal; GU, genito-urinary; N0, regional nodes not clinically involved by tumor; N1, regional nodes clinically involved by tumor; M0, no distant metastases; M1, distant metastases at diagnosis.

*Eligibility and quality control*

The following criteria have been used throughout all IRSG protocols. Newly diagnosed, previously untreated patients with RMS or UDS are eligible provided that they are less than 21 years of age at the commencement of therapy and are available for follow-up. In addition, therapy must be initiated within 42 days after the initial surgical procedure that provided diagnostic tissue. All pathologic materials are reviewed centrally, to ascertain eligibility. The IRSG surgeons review the operative procedures and information regarding grouping and staging. The IRSG radiation oncologists and the Quality Assurance Review Center (Providence, RI, USA) review all material related to XRT. The IRSG chemotherapists review the details of systemic treatment and assess protocol compliance. All of these reviews contribute to quality control.

### Results

The results of IRS-I to IRS-IV have been published.[1–7] The following presents the major lessons that have been learned as experience has accrued. These lessons are classified as surgical, radiotherapeutic, chemotherapeutic, and pathobiologic, and will be presented in that order.

*Surgery*

1. Patients with localized, completely resected disease (group I) generally have the best prognosis for 5-year failure-free survival (FFS) and overall survival. Patients with metastases at diagnosis (group IV) have the worst outlook, and those with group II and III disease have an intermediate prognosis. Thus, it has been preferable to try to remove all visible tumors, if feasible without excessive morbidity.
2. When a lesion has been excised without knowledge that it is malignant, wide re-excision is indicated, if feasible cosmetically and functionally, in order to obtain tumor-free margins.[9] This is particularly applicable to patients with primary tumor of the extremities.[10] Patients with group I ERMS do not need post-operative XRT.[11]
3. It is desirable to preserve organ function and thus spare such structures as the eye, vagina, and bladder. Furthermore, patients with tumor at or near these sites have a good prognosis. Primary chemotherapy followed by radiation therapy is the recommended approach. Delayed excision of initially unresected tumor may improve prognosis by changing a partial response into a complete response after initial shrinkage of the tumor by chemotherapy, with or without XRT.[12]
4. There is a relationship between age at diagnosis and likelihood of regional lymph node involvement in boys with non-metastatic paratesticular rhabdomyosarcoma. Event-free survival in IRS-IV was better for boys younger than 10 years of age, as the nodal relapse rate was lower than in those 10 years of age and older. We now recommend performing a modified ipsilateral retroperitoneal lymph-node dissection in older boys who have no clinical evidence of regional node involvement. If the nodes are uninvolved, cyclophosphamide and XRT are withheld; if tumor is present in the nodes, cyclophosphamide and XRT are given in addition to vincristine and actinomycin D.[13]

*Radiation therapy (XRT)*

1. There is no evidence to show benefit from giving radiation to patients with completely resected, localized lesions (group I), provided that the histologic subtype is ERMS.[11] Graded doses of irradiation are appropriate for all other patients, based on the patient's group at the time of study entry. Volumes to be irradiated include the pre-treatment primary tumor and regional lymph-nodal area, if involved. Patients with group IV disease receive XRT to both the primary site and to the sites of metastases, within the limits of bone-marrow tolerance.
2. A recent analysis of patients with group II disease in IRS-I to IRS-IV has shown improved outcome in IRS-III and IRS-IV, perhaps due to intensified therapy.[14]
3. Local failure rates for patients with group III disease in the IRS-III and IRS-IV studies have recently been reviewed. The rates have remained stable or improved. In IRS-IV, local failure rates were 2% in orbit primary sites, 16% in cranial parameningeal sites, and 12% in other head/neck sites. Local failure rates were 7% in extremity sites, 19% in genitourinary sites, and 14% in other sites.[15]
4. Thus far, there is no indication that giving hyperfractionated XRT to 59.4 Gy in two daily fractions of 1.1 Gy, with a 6-hour interfractional interval, will result in a better local-regional control rate among children with group III tumors than that obtained with 50.4 Gy in 1.8 fractions daily.[16]
5. Current IRSG results suggest that most patients with cranial parameningeal sarcoma, including those with localized intracranial extension in contiguity with the primary tumor at diagnosis, can be successfully managed with systemic chemotherapy and XRT. Radiation therapy is directed to the primary tumor, including any extension, along with a 2 cm margin, to include the adjacent meninges. Whole-brain XRT and intrathecal anticancer agents are not necessary in the absence of diffuse meningeal involvement or multiple intracranial metastases.[17]

*Chemotherapy*

1. Data from IRS-I, IRS-II, and IRS-III in 1431 patients indicate that there is no benefit from adding doxorubicin (DOX) to the combination of vincristine, actinomycin D, and cyclophosphamide (VAC) in patients with group III and IV disease, whether analyzed together or within group III and group IV categories individually.[1–3] The addition of DOX and cisplatin with or without etoposide to the VAC regimen has not improved outcome for patients with advanced disease in IRS-III.[3]

2. Data from IRS-IV indicate that the current standard combination of VAC, with cyclophosphamide at 2.2 g/m$^2$ per dose with GCSF is equally efficacious with regard to failure-free and overall survival as are VAI (vincristine, actinomycin D, and ifosfamide) and VIE (vincristine, ifosfamide, and etoposide).[7]
3. Escalation of cyclophosphamide dose from 0.9 g/m$^2$ in IRS-III to 2.2 g/m$^2$ in IRS-IV has improved the failure-free survival of patients with ERMS but not those with ARMS or UDS.[18]
4. Topotecan is a relatively new agent with the ability to disrupt topoisomerase I and thereby inhibit DNA replication. It is active in newly diagnosed patients with metastatic RMS, and can be given in combination with VAC.[19]

*Pathology and biology*

1. The results of the IRS-I and IRS-II studies indicated that patients with alveolar RMS have a worse outlook than those with embryonal RMS.[20,21] Treatment was then intensified, and outcome was improved for such patients in IRS-III. Many of the patients with ARMS are older patients with extremity primary tumors, both of which are unfavorable prognostic factors.[21] Patients with UDS also have a worse outlook than their counterparts with ERMS.[20,21]
2. In patients with ERMS of the bladder, the demonstration of maturing rhabdomyoblasts in sequential biopsies from the primary tumor after shrinkage following chemotherapy and radiation therapy does not necessarily signify the presence of malignant cells.[22] Thus, the current recommendations are not to use aggressive surgical interventions, but to continue chemotherapy and follow with repeated imaging studies along with biopsy when indicated, in order to preserve the bladder.
3. Molecular genetic studies have shown two consistent translocations in tumors from the majority of patients with ARMS. The t(2;13) translocation often occurs in older patients who have a worse outcome than their younger counterparts with the t(1;13) translocation. Members of this latter group are often infants who have a better prognosis than would be expected otherwise.[23,24] To date, there has been no consistently present translocation identified in ERMS.
4. Studies of the expression of P-glycoprotein[25] and of alterations in the p53 gene[26,27] may yield implications for the future therapy and prognosis of patients with RMS. It is possible that other substances and as yet undiscovered genetic changes will also have implications for directing future research in RMS. It is necessary to obtain fresh tumor samples at diagnosis to elucidate answers to basic biologic questions.
5. There is a small but appreciable incidence of second malignant neoplasms arising in children who have survived RMS.[28,29] The risk is highest in patients treated with both XRT and alkylating agents, especially melphalan.[30] Thus, all patients with RMS and UDS should be followed for many years to elucidate more precisely the incidence and proper management of this complication.

### The IRS-V Study

The IRS-V study combines group, stage, and histologic subtype to allocate patients to three different therapeutic protocols according to risk of recurrence. Low-risk patients have an estimated 3-year FFS rate of 88%; intermediate-risk patients have an estimated 3-year FFS rate of 55–76%, and high-risk patients have a 3-year FFS rate of < 30%. Multidisciplinary treatment is recommended as defined by histologic subtype and primary site, as well as the extent of disease at diagnosis and response to treatment. The goal is to achieve local control with preservation of form and function. The Appendix displays the elements of the protocols for the three risk groups and indicates therapy for each.

*Chemotherapy*

Low-risk patients have localized ERMS in favorable sites (stage 1) or in unfavorable sites (stages 2 and 3) that has been grossly completely removed, without (group I) or with (group II) microscopic residual disease and/or resected, tumor-involved regional lymph nodes. The patients with the best prognosis are placed in subgroup A and receive VA with or without XRT. The others, placed in subgroup B, received VAC ± XRT (see Appendix). Intermediate-risk patients have localized ARMS or UDS (stages 1–3) or ERMS (stages 2 and 3) with gross residual disease (group III), or ERMS with metastases (group IV) at < 10 years of age at diagnosis. They are randomized to receive VAC or VAC alternating with vincristine and cyclophosphamide plus topotecan, along with XRT. High-risk patients have ERMS at ≥ 10 years of age, or ARMS or UDS at any age < 21 years, with metastases at diagnosis (group IV). They receive a trial of irinotecan[31] over 6 weeks followed by VAC. Irinotecan is continued at intervals for those who have responded to it initially, but is omitted for non-responders. High-risk patients with cranial parameningeal tumors and meningeal impingement at diagnosis receive VAC without irinotecan.

### *Radiation therapy*

Patients with completely excised ERMS (i.e. group I) receive no XRT. However, patients with completely excised (group I) ARMS and UDS receive XRT to the primary site.[11] Other patients receive XRT as a function of group, histologic subtype and status of regional lymph nodes and/or distant metastases. Patients with metastases receive XRT to the primary tumor and to sites of metastases, within the limits of bone marrow tolerance.

### *Surgery*

The incidence of tumor-involved regional lymph nodes in patients with primary tumors of the extremity may be higher than initially suspected.[32] Sentinel lymph-node mapping, using a vital dye such as methylene blue along with radiolabelled technetium sulfur colloid, can localize the regional node most likely to contain tumor cells.[33] The surgeon can then remove the labeled node so that the pathologist can determine whether tumor cells are present. If they are, the node-bearing region should be irradiated. The utility of lymph-node mapping will be examined in IRS-V.

For patients whose tumors are initially deemed unresectable, a second-look procedure should be considered after initial chemotherapy. Recommended local control measures are specified by primary site.

### *Pathology and biology*

There is much to be learned about the biology of these tumors. Both fresh tissue and frozen tissue are necessary for ongoing studies and for new investigation in molecular biology.

### Acknowledgement

This manuscript is written with the support of NIH/NCI Grants CA-24507 and CA-72989.

### References

1 Maurer HM, Beltangady M, Gehan EA, *et al.* The Intergroup Rhabdomyosarcoma Study-I. A final report. *Cancer* 1988; 61:209–20.

2 Maurer HM, Gehan EA, Beltangady M, *et al.* The Intergroup Rhabdomyosarcoma Study-II. *Cancer* 1993; 71:1904–22.

3 Crist W, Gehan EA, Ragab AH, *et al.* The Third Intergroup Rhabdomyosarcoma Study. *J Clin Oncol* 1995; 13:610–30.

4 Ortega JA, Ragab AH, Gehan EA, *et al.* A feasibility, toxicity, and efficacy study of ifosfamide, actinomycin D, and vincristine for the treatment of childhood rhabdomyosarcoma: a report of the Intergroup Rhabdomyosarcoma Study IV Pilot Study. *Am J Pediatr Hematol Oncol* 1993; 15(suppl A):S15–20.

5 Ruymann FB, Vietti T, Gehan E, *et al.* Cyclophosphamide dose escalation in combination with vincristine and actinomycin D (VAC) in gross residual sarcoma: a pilot study without hematopoietic growth factor support evaluating toxicity and response. *J Pediatr Hematol Oncol* 1995; 17:331–7.

6 Arndt C, Tefft M, Gehan E, *et al.* A feasibility, toxicity, and early response study of etoposide, ifosfamide, and vincristine for the treatment of children with rhabdomyosarcoma: a report from the Intergroup Rhabdomyosarcoma Study (IRS) IV Pilot Study. *J Pediatr Hematol Oncol* 1997; 19:124–9.

7 Crist W, Anderson J, Maurer H, *et al.* Preliminary results for patients with local/regional tumors treated on the Intergroup Rhabdomyosarcoma Study-IV (1991–97) [abstract 2141]. *Proc Am Soc Clin Oncol* 1999; 18:555a.

8 Lawrence W Jr, Anderson JR, Gehan EA, *et al.* Pretreatment TNM staging of childhood rhabdomyosarcoma: a report of the Intergroup Rhabdomyosarcoma Study Group. *Cancer* 1997; 80:1165–70.

9 Hays DM, Lawrence W Jr, Wharam M, *et al.* Primary reexcision for patients with 'microscopic residual' tumor following initial excision of sarcomas of trunk and extremity sites. *J Pediatr Surg* 1989; 24:5–10.

10 Lawrence W Jr, Hays DM, Heyn R, *et al.* Surgical lessons from the Integroup Rhabdomyosarcoma Study (IRS) pertaining to extremity tumors. *World J Surg* 1988; 12:676–84.

11 Wolden SL, Anderson JR, Crist WM, *et al.* Indications for radiotherapy and chemotherapy after complete resection in rhabdomyosarcoma: a report from the Intergrup Rhabdomyosarcoma Studies I to III. *J Clin Oncol* 1999; 17:3468–75.

12 Wiener E, Lawrence W, Hays D, *et al.* Survival is improved in clinical group III children with complete response (CR) established by second-look operations in the Intergroup Rhabdomyosarcoma Study (IRS) III [abstract 228]. *Med Pediatr Oncol* 1999; 19:399.

13 Wiener ES, Anderson JR, Ojimba JI, *et al.* What is optimal management for children or adolescents with localized paratesticular rhabdomyosarcoma? — Results of IRS-III and IRS-IV. *J Pediatr Surg* (submitted).

14 Smith LM, Anderson JR, Qualman SJ, *et al.* Which patients with rhabdomyosarcoma (RMS) and microscopic residual tumor (Group II) fail therapy? A report from the Intergroup Rhabdomyosarcoma Study Group (IRSG) [abstract 2273B]. *Proc Am Soc Clin Oncol* 2000; 19:577a.

15 The IRSG Statistical Office, Personal Communication, 14 August 2000.

16 Donaldson SS, Meza JL, Breneman J, *et al.* Results from the Intergroup Rhabdomyosarcoma Study-IV randomized trial of hyperfractionated radiation in children with rhabdomyosarcoma [abstract 132]. *Int J Radiat Oncol Biol Phys* 2000; 48(Suppl.):178.

17 Raney B, Meza J, Fryer C, *et al.* Results of treating localized cranial parameningeal sarcoma on Intergroup Rhabdomyosarcoma (RMS) Studies (IRS)-II through -IV, 1978–1997 [abstract O-36]. *Med Pediatr Oncol* 2000; 35:178.

18 Anderson JR, Link M, Qualman S, *et al.* Improved outcome for patients (pts) with embryonal (emb) histology (hist) but not alveolar hist rhabdomyosarcoma (RMS): results from Intergroup Rhabdomyosarcoma Study IV (IRS-IV) [abstract 2022]. *Proc Am Soc Clin Oncol* 1998; 17:526a.

19 Pappo AS, Lyden E, Breneman J, *et al.* Up-front window trial of topotecan in previously untreated children and adolescents with metastatic rhabdomyosarcoma: an Intergroup Rhabdomyosarcoma Study (IRSG). *J Clin Oncol* 2001; 19:213–9.

20 Newton WA Jr, Soule EH, Hamoudi AB, *et al.* Histopathology of childhood sarcomas, Intergroup

Rhabdomyosarcoma Studies I and II; clinicopathologic correlations. *J Clin Oncol* 1988; 6:67–75.

21 Crist WM, Garnsey L, Beltangady MS, *et al.* Prognosis in children with rhabdomyosarcoma: a report of the Intergroup Rhabdomyosarcoma Studies I and II. *J Clin Oncol* 1990; 8:443–52.

22 Heyn R, Newton WA, Raney RB, *et al.* Preservation of the bladder in patients with rhabdomyosarcoma. *J Clin Oncol* 1997; 15:69–75.

23 Barr FG. Molecular genetics and pathogenesis of rhabdomyosarcoma. *J Pediatr Hematol/Oncol* 1997; 19:483–91.

24 Kelly KM, Womer RB, Sorensen PHB, Xiong Q-B, Barr FG. Common and variant gene fusions predict distinct clinical phenotypes in rhabdomyosarcoma. *J Clin Oncol* 1997; 15:1831–6.

25 Kuttesch JF, Parham DM, Luo X, *et al.* P-Glycoprotein expression at diagnosis may not be a primary mechanism of therapeutic failure in childhood rhabdomyosarcoma. *J Clin Oncol* 1996; 14:886–900.

26 Malkin D, Li FP, Strong LC, *et al.* Germ line mutations in a familial syndrome of breast cancer, sarcomas, and other neoplasms. *Science* 1990; 250:1233–8.

27 Strong LC, Williams WR, Tainsky MA. The Li–Fraumeni syndrome: from clinical epidemiology to molecular genetics. *Am J Epidemiol* 1992; 135:190–9.

28 Heyn R, Haeberlen V, Newton WA, *et al.* Second malignant neoplasms in children treated for rhabdomyosarcoma. *J Clin Oncol* 1993; 11:262–70.

29 Heyn R, Khan F, Ensign LG, *et al.* Acute myeloid leukemia in patients treated for rhabdomyosarcoma with cyclophosphamide and low-dose etoposide on Intergroup Rhabdomyosarcoma Study III: an interim report. *Med Pediatr Oncol* 1994; 23:99–106.

30 Pappo A, Anderson J, Qualman S, Donaldson S, Crist W. Second malignant neoplasms (SMN) in IRSG-IV: a preliminary report from the Intergroup Rhabdomyosarcoma Study Group (IRSG) [abstract 2298]. *Proc Am Soc Clin Oncol* 2000;19:584a.

31 Furman W, Stewart C, Pratt C, *et al.* A Phase I study of irinotecan (CPT-11) in children with relapsed solid tumors [abstract 721]. *Proc Am Soc Clin Oncol* 1998; 17:187a.

32 Mandell L, Ghavimi F, LaQuaglia M, Exelby P. Prognostic significance of regional lymph node involvement in childhood extremity rhabdomyosarcoma. *Med Pediatr Oncol* 1990; 18:466–71.

33 Neville HL, Andrassy RJ, Lally KP, *et al.* Lymphatic mapping with sentinel node biopsy in pediatric patients. *J Pediatr Surg* 2000; 35:961–4.

Appendix: IRSG Study V risk assignments

| Risk (protocol) | Stage | Group | Site* | Size† | Age (years) | Histology‡ | Metastasis** | Nodes†† | Treatment‡‡ |
|---|---|---|---|---|---|---|---|---|---|
| Low, subgroup A | 1 | I | Favorable | a or b | < 21 | EMB | M0 | NO | VA |
| (D9602) | 1 | II | Favorable | a or b | < 21 | EMB | M0 | NO | VA + XRT |
| | 1 | III | Orbit only | a or b | < 21 | EMB | M0 | NO | VA + XRT |
| | 2 | I | Unfavorable | a | < 21 | EMB | M0 | NO or NX | VA |
| Low, subgroup B | 1 | II | Favorable | a or b | < 21 | EMB | M0 | N1 | VAC + XRT |
| (D9602) | 1 | III | Orbit only | a or b | < 21 | EMB | M0 | N1 | VAC + XRT |
| | 1 | III | Favorable (excluding orbit) | a or b | < 21 | EMB | M0 | NO or N1 or NX | VAC + XRT |
| | 2 | II | Unfavorable | a | < 21 | EMB | M0 | NO or NX | VAC + XRT |
| | 3 | I or II | Unfavorable | a | < 21 | EMB | M0 | N1 | VAC (+ XRT, Gp II) |
| | 3 | I or II | Unfavorable | b | < 21 | EMB | M0 | NO or N1 or NX | VAC (+ XRT, Gp II) |
| Intermediate | 2 | III | Unfavorable | a | < 21 | EMB | M0 | N0 or NX | VAC ± Topo + XRT |
| (D9803) | 3 | III | Unfavorable | a | < 21 | EMB | M0 | N1 | VAC ± Topo + XRT |
| | 3 | III | Unfavorable | b | < 21 | EMB | M0 | N0 or N1 or NX | VAC ± Topo + XRT |
| | 1 or 2 or 3 | I or II or III | Favorable or unfavorable | a or b | < 21 | ALV/UDS | M0 | N0 or N1 or NX | VAC ± Topo + XRT |
| | 4 | I or II or III or IV | Favorable or unfavorable | a or b | < 10 | EMB | M1 | N0 or N1 or NX | VAC ± Topo + XRT |
| High | 4 | IV | Favorable or unfavorable | a or b | ≥ 10 | EMB | M1 | N0 or N1 or NX | CPT-11, VAC + XRT |
| (D9802) | 4 | IV | Favorable or unfavorable | a or b | < 21 | ALV/UDS | M1 | N0 or N1 or NX | CPT-11, VAC + XRT |

* Favorable, orbit/eyelid, head and neck (excluding parameningeal), genito-urinary (not bladder or prostate); biliary tract; unfavorable, bladder, prostate, extremity, parameningeal, trunk, retroperitoneal, pelvis, other.

† a, Tumor size ≤ 5 cm in diameter; b, tumor size > 5 cm in diameter.

‡ EMB, Embryonal, botryoid or spindle-cell rhabdomyosarcomas or ectomesenchymonas with embryonal RMS; ALV, alveolar rhabdomyosarcomas, or ectomesenchymomas with alveolar RMS; UDS, undifferentiated sarcomas.

** M0, No distant metastases; M1, distant metastases at diagnosis.

†† N0, Regional nodes clinically not involved; N1, regional nodes clinically involved; NX, node status unknown.

‡‡ VAC, Vincristine, actinomycin D, cyclophosphamide; XRT, radiotherapy; Topo, topotecan; Gp, group; CPT-11, irinotecan.
